# Tumeur évoluée du tronc d’origine lymphomateuse

**DOI:** 10.11604/pamj.2018.30.57.15119

**Published:** 2018-05-24

**Authors:** Mouna Ejjiyar, Saloua Ettalbi

**Affiliations:** 1 Service de Chirurgie Plastique, CHU Mohammed VI, Marrakech, Maroc

**Keywords:** Lymphome malin, tumeur évoluée, localisation cutanée, Malignant lymphomas, advanced tumor, cutaneous localization

## Abstract

Malignant lymphomas are a group of cancers that arise in the lymphoid tissue, in the lymph nodes or in extranodal sites due to neoplastic lymphocytic cell transformation. Within this group, malignant non-Hodgkin lymphomas are distinctive. We report a case of diffuse advanced and extremely aggressive malignant cutaneous non-Hodgkin lymphoma. The study involved a 30-year old patient, driver, married and father of one child, followed-up in the Department of Hematology and Oncology for 2 years due to malignant non-Hodgkin lymphoma diagnosed on the basis of axillary and inguinal polyadenopathies. The patient underwent 13 chemotherapy sessions. He was admitted to the Department of Plastic, Cosmetic and Burn Surgery at the University Hospital Mohammed VI in Marrakech with skin involvement of his lymphoma manifesting as an advanced left latero-thoracic tumor rapidly increasing in size. On clinical examination, his general condition was quite good. It showed voluminous left latero-thoracic mass adherent to its deeper structure with ipsilateral axillary shield. Partial biopsy was performed. Anatomo-pathological examination confirmed the diagnosis of diffuse malignant large B-cells non-Hodgkin’s lymphoma. Immunohistochemical and genetic study was not performed. Serology tests were negative. Serial Computed Tomography (CT) scan showed voluminous left antero-lateral predominantly axillary and thoracic ganglionic mass made of confluent lymph nodes, with persistence of peripheral lymph nodes, skin ulcers and necrotic areas measuring 30cm in diameter. In our Hospital, the patient underwent wide tumor resection, then coverage with a split-thickness skin, thus reducing tumor volume and ensuring better patient’s comfort. When the patient started healing, he was referred to the Department of Hematology and Oncology for complementary treatment.

## Image en médecine

Les lymphomes malins constituent un groupe de cancers du tissu lymphoïde, ganglionnaire ou extra ganglionnaire, liés à la transformation néoplasique d'une cellule lymphocytaire. On distingue dans cette catégorie les lymphomes malins non hodgkiniens. Nous rapportons le cas d'une localisation cutanée évoluée et extrêmement agressive d'un lymphome malin non hodgkinien diffus. Il s'agit d'un patient âgé de 30 ans, marié et père d'un enfant, chauffeur de profession, suivi en hémato-oncologie depuis 2 ans pour lymphome malin non hodgkinien diagnostiqué devant des poly adénopathies axillaires et inguinales, ayant bénéficié de 13 séances de chimiothérapie. Le patient a été admis au service de chirurgie plastique, réparatrice, esthétique et des brûlés du CHU Mohammed VI de Marrakech, pour prise en charge d'une localisation cutanée de son lymphome sous la forme d'une tumeur évoluée de la région latéro-thoracique gauche augmentant rapidement de taille. A l'examen, on retrouvait un patient en assez bon état général, présentant une volumineuse masse latéro-thoracique gauche, adhérente à son plan profond avec blindage axillaire homolatéral. Une biopsie partielle réalisée a confirmé à l'examen anatomo-pathologique le diagnostic de lymphome malin non hodgkinien diffus à grandes cellules. L'étude immunohistochimique et génétique n'a pas été réalisée. Les sérologies étaient négatives. L'imagerie a permis de révéler sur les coupes tomodensitométriques la présence d'une volumineuse masse ganglionnaire à prédominance axillaire et thoracique antéro-latérale gauche mesurant 30cm de diamètre, faite d'une confluence de ganglions avec persistance de ganglions nodulaires en périphérie, ulcérations cutanées et zones de nécrose. Le patient a bénéficié dans notre structure d'une exérèse tumorale large de propreté puis d'une couverture par de la peau mince, réduisant ainsi le volume tumoral et assurant un meilleur confort pour le patient, qui fut adressé dès cicatrisation en hémato-oncologie pour bénéficier de son traitement complémentaire.

**Figure 1:**
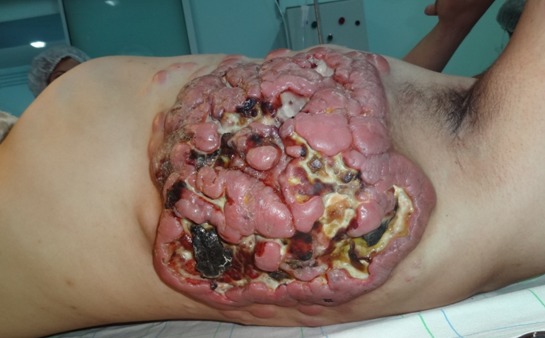
Localisation cutanée d’un lymphome malin

